# Epigenetic Variation Induced by Gamma Rays, DNA Methyltransferase Inhibitors, and Their Combination in Rice

**DOI:** 10.3390/plants9091088

**Published:** 2020-08-24

**Authors:** Sung-Il Lee, Jae Wan Park, Soon-Jae Kwon, Yeong Deuk Jo, Min Jeong Hong, Jin-Baek Kim, Hong-Il Choi

**Affiliations:** Advanced Radiation Technology Institute, Korea Atomic Energy Research Institute, Jeongeup 56212, Korea; lsi@kaeri.re.kr (S.-I.L.); jwpark273@kaeri.re.kr (J.-W.P.); soonjaekwon@kaeri.re.kr (S.-J.K.); jyd@kaeri.re.kr (Y.D.J.); hongmj@kaeri.re.kr (M.J.H.); jbkim74@kaeri.re.kr (J.-B.K.)

**Keywords:** DNA methylation, epigenetic diversity, DNA methyltransferase inhibitors, gamma rays, methylation-sensitive amplified polymorphism, transposon methylation display

## Abstract

DNA methylation plays important roles in the regulation of gene expression and maintenance of genome stability in many organisms, including plants. In this study, we treated rice with gamma rays (GRs) and DNA methyltransferase inhibitors (DNMTis) to induce variations in DNA methylation and evaluated epigenetic diversity using methylation-sensitive amplified polymorphism (MSAP) and transposon methylation display (TMD) marker systems. Comparative and integrated analyses of the data revealed that both GRs and DNMTis alone have epimutagenic effects and that combined treatment enhanced these effects. Calculation of methylation rates based on band scoring suggested that both GRs and DNMTis induce epigenetic diversity by demethylation in a dose-dependent manner, and combined treatment can induce variations more synergistically. The difference in the changes in full and hemi-methylation rates between MSAP and TMD is presumed to be caused by the different genomic contexts of the loci amplified in the two marker systems. Principal coordinate, phylogenic, and population structure analyses commonly yielded two clusters of individuals divided by DNMTi treatment. The clustering pattern was more apparent in TMD, indicating that DNMTis have a stronger effect on hypermethylated repetitive regions. These findings provide a foundation for understanding epigenetic variations induced by GRs and DNMTis and for epigenetic mutation breeding.

## 1. Introduction

DNA methylation contributes to the epigenetic regulation of gene expression, genomic stability, and transposon silencing and plays important roles in multiple biological processes, including plant and mammalian development [1,2]. DNA methylation has been a focus of recent research that has sought to identify beneficial new traits, and it has been established that hypermethylation is generally correlated with the downregulation of gene expression, whereas hypomethylation is correlated with upregulation [3,4]. DNA methylation maintains the methylation state after a methyl group has been added via the action of DNA methyltransferases and may also occur passively when methylation does not occur in the newly synthesized DNA strand during DNA replication [5,6]. Additionally, 5-methylcytosine can be actively removed by the base excision repair pathway [2]. The chemical inhibition of DNA methyltransferases using DNA methyltransferase inhibitors (DNMTis), such as 5-azacytidine (AZA) and zebularine (ZEB), as demethylating agents is an approach used to study the effects of DNA methylation loss in plants [7,8]. Although AZA and ZEB have similar inhibitory effects on DNA methylation, ZEB has a longer half-life and better stability than AZA [8,9]. Many studies have investigated the effects of DNMTis on gene expression and the silencing, growth, development, and induction of DNA damage in plants [10,11,12,13].

Transposable elements (TEs) are ubiquitous in the genomes of eukaryotes, and their relocalization within genomes serves to generate genomic plasticity by inducing various types of genic and intergenic mutations that can increase allelic diversity [14,15,16]. After being discovered first by McClintock in the 1940s, TEs were initially considered junk or selfish DNA [17,18]. However, they are now recognized to play important roles as a driving force of evolution in eukaryotic taxa [19]. TEs can be classified into two types based on their mechanism of transposition: class I retrotransposable elements and class II DNA transposons. Class I retrotransposons make new copies in different chromosomal locations via a “copy-and-paste” mechanism of transposition, whereas class II DNA transposons move via a “cut-and-paste” mechanism [20]. Transposition activity, including the movement of DNA transposons or the insertion of new copies of retrotransposons in a genome, can affect genomic stability [21]. Accordingly, TEs tend to be hypermethylated relative to other genomic sequences in eukaryotic genomes [2,22,23]. In *Arabidopsis*, methylation occurs at high levels in heterochromatin and euchromatin, including in some transposons, and this high level of DNA methylation seems to inhibit transposon mobility [23]. A high methylation rate in TEs and adjacent genomic regions was also reported in rice [24].

Molecular marker systems are useful tools that can be used in various fields, such as taxonomy, plant breeding, and genetic engineering, to assess genetic diversity, identify and fingerprint genotypes, determine genetic distances between populations, strains, and breeding materials, and be the basis of marker-assisted selection [25,26,27]. Amplified fragment length polymorphism (AFLP), one of the most popular PCR-based marker systems, uses two sticky-end restriction enzymes and ligate adapters to detect mutations in restriction sites dispersed throughout genomes. Methylation-sensitive amplified polymorphism (MSAP) is a modified AFLP method used to detect DNA methylation polymorphisms using methylation-sensitive *Hpa*II and its methylation-insensitive isoschizomer *Msp*I, and both enzymes recognize 5′-CCGG-3′ sequences [28,29]. Transposon methylation display (TMD) is a modified MSAP method that uses an additional primer specific to transposons to identify methylation patterns at CCGG sites [4].

Rice is an important cereal crop for human consumption, with over 100,000 cultivars worldwide [30]. It is a diploid species with a small genome and has become the most well-studied crop with respect to functional genomics, as it is used as a monocot model plant [31,32]. Various methods of mutation breeding have been developed to identify and improve rice characteristics, and extensive studies have been conducted on mutagenesis using physical and chemical agents [31]. In terms of physical mutagens, gamma rays (GRs) have been used in the development of more than 90% of rice varieties, in addition to X-rays, neutrons, and protons [33,34,35]. Regarding chemical mutagens, studies using ethyl methane sulfonate [36], sodium azide [37], and *N*-methyl-*N*-nitrosourea [38] have been reported. Furthermore, the efficiency, effectiveness, and induced chlorophyll mutation rate of combined treatment with GRs and ethyl methane sulfonate have been reported [36,39,40,41]. To date, however, there have been no studies that have examined the effects of combined treatment with GRs and DNMTis on epigenetic mutations.

Here, we investigated methylation variation in rice plants generated from seeds subjected to treatment with GRs, DNMTis, and their combination by MSAP and TMD analyses. The enzyme combinations *Hpa*II/*Mse*I and *Msp*I/*Mse*I were used for MSAP analysis. A *p-SINE1* retrotransposon-specific primer with *Hpa*II/*Msp*I adapters was adopted for TMD analysis.

## 2. Results

### 2.1. Evaluation of Polymorphisms Detected by MSAP and TMD Marker Systems

Six and four primer combinations were used for MSAP and TMD analyses, respectively (see details in Materials and Methods). Each primer combination generated 10 to 40 discernable loci ranging in size from 150 to 500 bp. Ultimately, MSAP and TMD yielded 102 and 60 loci, 66 and 41 of which were polymorphic, respectively (Table 1). All indices related to polymorphism level, including the percentage of polymorphic loci, Nei’s gene diversity (*H*), and Shannon’s information index (*I*), were higher in TMD than in MSAP (Table 1).

### 2.2. Methylation Changes Induced by Treatment with GRs and DNMTis

Methylation levels and patterns in rice treated with GRs and DMNTis were estimated based on the band scoring of individual plants. In the MSAP analysis, the total methylation rates in the control (58.69%) and GR-irradiated groups (100 Gy [G100], 58.42%; 150 Gy [G150], 58.80%; and 250 Gy [G250], 57.43%) were similar, whereas the DNMTi (AZA 80 µM [A80], 54.91%; ZEB 80 µM [Z80], 54.35%) and combined (G100 + A80, 53.94%; G150 + A80, 54.04%; G100 + Z80, 54.66%; and G150 + Z80, 52.63%) treatment groups showed lower rates (Table 2). The combined treatment group showed the most reduced methylation rate (average 4.87%) relative to the control (Table 2). Both the full and hemi-methylation rates were lower in all treatment groups than in the control, although this was more apparent in the DMNTi and combined treatment groups (Table 2).

The TMD analysis showed different DNA methylation patterns than the MSAP analysis (Table 2 and Table 3). The total methylation rates were similar between the control and GR and DMNTi treatment groups, whereas the total methylation rate in the combined treatment group was 6.66% lower on average. Interestingly, both the full and hemi-methylation rates shown by TMD were distinctly different from those shown by MSAP. The full methylation rate was highest in the control group (23.35%), followed by the GR-irradiated group (average 14.06%), the DNMTi treatment group (average 12.66%), and the combined treatment group (average 7.56%). By contrast, the hemi-methylation rate was lowest in the control group (7.05%), followed by the GR-irradiated group (average 12.13%), the DNMTi treatment group (average 16.46%), and the combined treatment group (average 21.38%).

### 2.3. Analysis of Molecular Variance

We performed analysis of molecular variance (AMOVA) in order to determine whether the epigenetic variations observed among rice groups treated with either GRs or DNMTis were differentiated from each other. The MSAP and TMD data showed that 74% and 67% of the variances could be attributed to differences within groups, respectively (Table 4). The variance among groups shown by TMD (33%) was higher than that shown by MSAP (26%), indicating that higher epiallele diversity was induced in the loci amplified in TMD. The AMOVA result for the total data (MSAP and TMD data combined) showed that the variance within groups and among groups was 71% and 29%, respectively. The correlation coefficient between the epigenetic distance of the two marker systems was calculated using the Mantel test, which revealed that there was no correlation (*r*^2^ = 0.1612).

### 2.4. Population Structure and Phylogenetic Analysis

**Figure 1:**
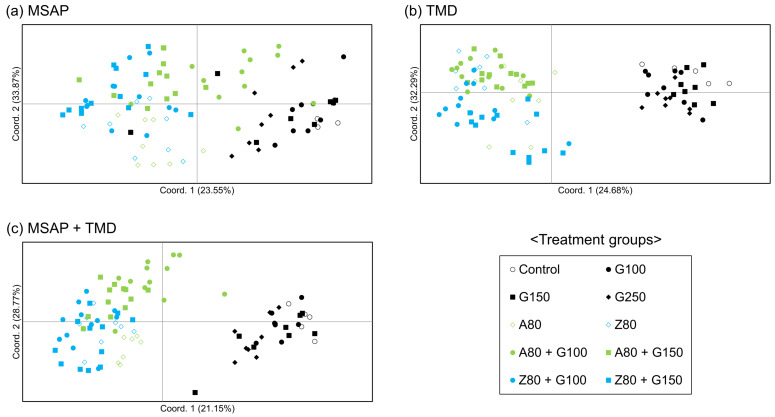
Principal coordinate analyses (PCoA) of rice treated with gamma rays (GRs) and DNA methyltransferase inhibitors (DNMTis) based on methylation-sensitive amplified polymorphism (MSAP) and transposon methylation display (TMD). PCoA plots for (**a**) MSAP, (**b**) TMD, and (**c**) combined MSAP and TMD data.

Principal coordinate analyses (PCoA) were performed to investigate the epigenetic relationships among the different treatment groups (Figure 1). In the PCoA plot for MSAP, the control and GR-irradiated groups were distributed closely, and some individuals were located closer to those in the DNMTi and combined treatment groups, which showed a scattered distribution (Figure 1a). The PCoA plot for TMD showed two distinct clusters: a cluster of the control and GR-irradiated groups and another cluster of the DNMTi and combined treatment groups (Figure 1b). In other words, these two clusters were divided whether the individuals were treated with DNMTis or not. This trend was similar in the PCoA plot for merged MSAP and TMD data, although a few individuals were separated from the clusters (Figure 1c).

**Figure 2:**
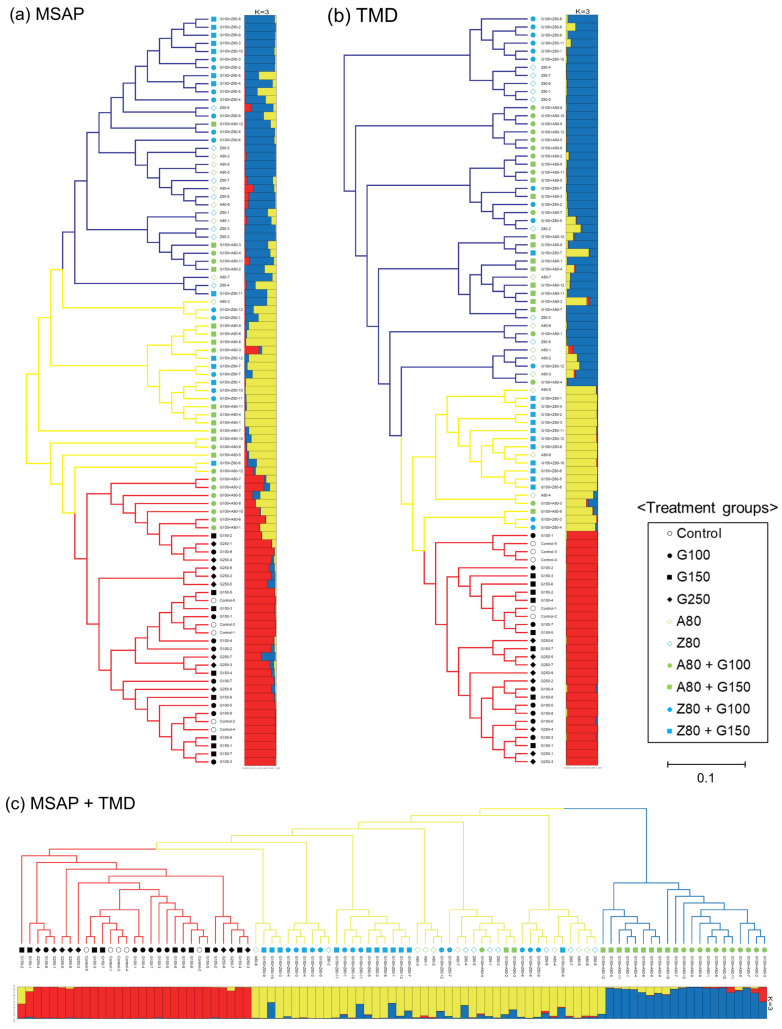
Phylogenetic relationships and population structure of rice treated with gamma rays (GRs) and DNA methyltransferase inhibitors (DNMTis) based on methylation-sensitive amplified polymorphism (MSAP) and transposon methylation display (TMD). PCoA plots of (**a**) MSAP, (**b**) TMD, and (**c**) combined MSAP and TMD data. The control and GR-irradiated groups formed one cluster (red branches), and the DNMTi and combined treatment groups formed two intermingled clusters (yellow and blue branches).

Population structure and phylogenetic relationships were analyzed based on the MSAP and TMD data. The calculation of delta *K* suggested that the optimal number of clusters (*K*) was three for all MSAP, TMD, and merged data (Figure S1). The three phylogenetic trees showed similar clustering trends: the control and GR-irradiated groups formed one cluster (depicted by red branches in Figure 2) and the DNMTi and combined treatment groups formed two intermingled clusters (depicted by yellow and blue branches in Figure 2). There were some exceptions: seven plants in the combined treatment group (A80 + G100) were found in the MSAP tree. However, these exceptions were not notable because they clustered outside of the main cluster with different epigenetic properties in the MSAP tree, and they were distributed far from the clusters of the control and GR-irradiated groups in both the TMD and merged data trees. The three clusters (*K* = 3) were therefore clearly divided according to data source.

## 3. Discussion

Methylation changes in plant DNA are molecular mechanisms that confer adaptations to various environmental factors, thus contributing to biodiversity [43,44]. In the present study, we found that epigenetic variations were induced by GRs, DNMTis, and their combination in rice plants based on MSAP and TMD analyses. Indices related to epigenetic diversity were increased in all treatment groups compared to the control (Table 1), indicating that GRs as well as DNMTis can affect DNA methylation status. The two types of DNMTi used here, AZA and ZEB, induce epigenetic variation by reducing the methylation rate [13]. Ionizing irradiation is also known to have a demethylating effect, which was deduced from the activation of TEs by hypomethylation in irradiated plants [33,45]. Low-energy ion beam irradiation induced both methylation and demethylation in *Arabidopsis* [46]. Together, the results of the present study indicate that both GRs and DNMTis play important roles in increasing epigenetic diversity and that combined treatment can induce more methylation variations.

The total methylation rates, calculated based on banding patterns, were reduced in all treatment groups (Table 2 and Table 3). As the treatment dose became higher, the MSAP data showed decreases in both the full and hemi-methylation rates (Table 2), whereas the TMD data showed a decrease in the full methylation rate and an increase in the hemi-methylation rate. This may be explained by the different genomic context of the loci amplified in each marker system. DNA methylation in plant genomes is distributed mainly in repetitive regions to stabilize genomes by inhibiting the activation of TEs [2,22,47]. The short interspersed retroelement *p-SINE1* used in this study for TMD, which was first identified in rice, is dispersed across the genome with a high copy number (approximately 6500 copies per haploid genome) [48]. This retroelement is a repetitive sequence of 70–500 bp with an internal promoter for RNA polymerase III, which is involved in transcription, although it lacks open reading frames [48,49]. A high level methylation of *p-SINE1* has been reported not only within the retroelement itself but also in adjacent regions in the rice genome [24]. Therefore, the increase in hemi-methylation shown by TMD may be a result of fully methylated repeat-rich regions being partially demethylated by treatment with GRs and DNMTis, which converted fully methylated loci into hemi-methylated loci.

AMOVA revealed that a large proportion of the epigenetic diversity observed in the present study was due to within-group variations (Table 4). This suggests that the methylation variations induced by GRs and DNMTis occur almost randomly. However, a higher percentage of variation among groups was observed in the TMD data than in the MSAP data (Table 4), indicating that the amplified loci in TMD, which may be highly methylated, were more dynamically affected by treatment with different doses of GRs and DNMTis. The differential influence on methylation variation between GRs and DNMTis could be inferred from the PCoA and population structure analysis (Figure 1 and Figure 2). The two clusters of rice plants clearly divided by DNMTi treatment in the PCoA plot (Figure 1b) reflect the fact that demethylation induced by DNMTis can produce more epigenetic diversity in hypermethylated repetitive regions. This tendency is similarly observed in the TMD-based phylogenetic tree and its population structure (Figure 2b). The intermixed cluster containing the DNMTi and combined treatment groups branches from the cluster of the control and GR-irradiated groups, which reflects increased methylation variation due to demethylation by DNMTis.

In this study, we revealed methylation variation induced by GRs and DNMTis in rice, which can be synergistically increased when the two mutagens are administered in combination. Epigenetic diversity is potentially of value for crop breeding, including for widening the range of phenotypic variation, increasing stress tolerance with less gene erosion, and balancing major agronomic traits in a species [50]. Although the question of whether epigenetic changes can be transmitted to progeny has remained controversial, a great deal of experimental evidence of transgenerational epigenetic inheritance has been reported, particularly with regard to DNA methylation [51]. The present study was conducted only on M_1_ plants, so further investigation into subsequent generations is needed. The findings of this study will lay the foundation for understanding the variations in DNA methylation artificially induced by GRs, DNMTis, and their combination.

## 4. Materials and Methods

### 4.1. Plant Materials and Genomic DNA Extraction

Solutions of two types of DNMTi, AZA (Sigma-Aldrich, St Louis, MO, USA) and ZEB (TCI, Tokyo, Japan), were prepared in 0.5% dimethyl sulfoxide (DMSO; Sigma-Aldrich, St Louis, MO, USA). Rice (*Oryza sativa* L. ssp. *japonica* cv. Ilpum) seeds were soaked in 80 µM DNMTi solution for 24 h at room temperature. Seeds in the control group (no treatment with either GRs or DNMTis) and the GR irradiation only group were pre-soaked in 0.5% DMSO solution for 24 h at room temperature. Seeds were irradiated using a ^60^Co gamma irradiator at the Korea Atomic Energy Research Institute (Jeongeup, Republic of Korea) for 8 h at a dose of 100, 150, or 250 Gy (dose rates of 12.5, 18.75, and 31.25 Gy h^−1^, respectively). The DNMTi solution treatment dose and time (80 µM for 24 h, respectively) were determined in our preliminary study, in which rice treated at this dose for this length of time had a survival rate of approximately 70%. In the combined treatment, irradiation doses of 100 and 150 Gy were applied because plants from seeds treated with DNMTi and ≥200 Gy GR irradiation did not survive after emergence.

Young leaves were harvested from plants during the three-leaf stage, and genomic DNA was extracted using a DNeasy Plant Mini Kit (Qiagen, Germantown, MD, USA). Quantification of the extracted DNA was performed using a NanoDrop spectrophotometer, and DNA integrity was assessed based on 1.0% agarose gel electrophoresis. Aliquots of the quantified total genomic DNA were subsequently diluted to a final concentration of 500 ng μL^−1^.

### 4.2. Molecular Marker Analysis

The MSAP analysis was conducted according to Reyna-López et al. [52]. Genomic DNA (500 ng) was digested for 16 h at 37 °C with 5 U of each restriction enzyme combination (*Mse*I/*Msp*I and *Mse*I/*Hpa*II) in 10 µL of buffer solution (Invitrogen, Carlsbad, CA, USA). The digested DNA was ligated in a final reaction volume of 25 µL with 15 pmol enzyme adapters (listed in Table S1) using T4 DNA ligase (Invitrogen, Carlsbad, CA, USA) for 16 h at 20 °C and diluted 1:4 with Tris-EDTA (TE) buffer. The ligated DNA (3 µL) was amplified with 10 pmol pre-amplification primers in a final reaction volume of 25 µL. The PCR conditions for pre-amplification were as follows: 95 °C for 2 min, followed by 20 cycles of 95 °C for 30 s, 55 °C for 30 s, and 72 °C for 1 min, and a final extension at 72 °C for 5 min. The pre-amplified product was diluted 1:100 with TE buffer, and 3 µL were used for selective amplification with the MSAP primer combinations listed in Table 4 under the following PCR conditions: 95 °C for 2 min, followed by 25 cycles of 95 °C for 30 s, 55 °C for 30 s, and 72 °C for 1 min, and a final extension at 72 °C for 5 min. The PCR products were diluted 1:4 with TE buffer and segregated using a Fragment Analyzer 5300 (Agilent, Santa Clara, CA, USA).

TMD analyses with *p-SINE*1 were carried out according to the protocol described by Takata et al. [24], with minor modifications. DNA digestion, pre- and selective amplification, and segregation of PCR products were carried out under the same conditions as MSAP with the TMD primer combinations listed in Table S2.

### 4.3. Data Analysis

DNA bands between 150 and 600 bp were scored as either present (1) or absent (0), and indistinct bands were excluded to avoid potential genotyping errors. POPGENE version 3.2 [53] was used to calculate the polymorphic index, Nei’s genetic diversity (*H*), Shannon’s information index, and the effective number of alleles. A genetic diversity matrix was produced using GenAlEx version 6.5 [54] for Mantel’s tests, PCoA, and AMOVA. Genetic distances and similarity coefficients were calculated using PowerMarker version 3.25 [55]. The neighbor-joining clustering method was used to construct an unrooted phylogenetic tree of rice individuals using MEGA X [56]. The population structure was analyzed based on Bayesian inference using STRUCTURE version 2.3.4 [57]. Calculations of *K* values from 1 to 10 were repeated 10 times to obtain log Pr (*X*|*K*) values. STRUCTURE HARVESTER web version 0.6.94 was used to estimate the optimal value of *K* [58].

## Figures and Tables

**Table 1:** Summary statistics of methylation-sensitive amplified polymorphism (MSAP) and transposon methylation display (TMD) marker systems in this study.

Marker Type and Groups	No. of Samples	No. of Primer Sets	No. of Loci	No. of Polymorphic Loci (%)	Nei’s Gene Diversity (*H*)	Shannon’s Information Index (*I*)	PIC ^1^
MSAP							
Control	5	6	102	23 (22.55)	0.292	0.433	
GR ^2^	24	6	102	41 (40.20)	0.341	0.498	
DNMTi ^3^	16	6	102	39 (38.24)	0.357	0.538	
DNMTi + GR ^4^	48	6	102	61 (59.80)	0.366	0.549	
Total	93	6	102	66 (64.71)	0.340	0.500	0.63
TMD							
Control	5	4	60	15 (25.00)	0.438	0.627	
GR ^2^	24	4	60	24 (40.00)	0.465	0.657	
DNMTi ^3^	16	4	60	29 (48.33)	0.484	0.677	
DNMTi + GR ^4^	48	4	60	34 (56.67)	0.497	0.679	
Total	93	4	60	41 (68.33)	0.420	0.660	0.65

^1^ Polymorphic information content. ^2^ Gamma ray (GR)-irradiated group. ^3^ DNA methyltransferse inhibitor (DNMTi) treatment group. ^4^ Combined treatment group with DNMTis and GRs.

**Table 2:** DNA methylation patterns in rice treated with gamma rays and DNA methyltransferase inhibitors detected by methylation-sensitive amplified polymorphism.

Band Type	M ^1^	H ^1^	Treatment Groups									
			Control	G100	G150	G250	A80	Z80	A80 + G100	A80 + Z150	Z80 + G100	Z80 + G150
I	1	1	221	336	337	338	363	362	573	580	550	586
II	1	0	70	100	67	84	57	59	90	92	96	102
III	0	1	102	126	97	84	54	48	83	78	82	46
IV	0	0	142	246	317	288	331	324	498	512	485	503
Total methylated (%) ^2,3^			58.69	58.42	58.8	57.43	54.91	54.35	53.94	54.04	54.66	52.63
Fully methylated (%) ^2,4^			13.08	12.38	8.19	10.58	7.08	7.44	7.23	7.29	7.91	8.25
Hemi-methylated (%) ^2,5^			19.07	15.59	11.86	10.58	6.71	6.05	6.67	6.18	6.76	3.72
Non-methylated (%)^2,6^			41.31	41.58	41.2	42.57	45.09	45.65	46.06	45.96	45.34	47.37

^1^ M and H indicate the enzyme combinations *Mse*I/*Msp*I and *Mse*I/*Hpa*II; 0: band absent, 1: band present. ^2^ Methylation rate was calculated according to Choi et al. 2016 [42]. ^3^ Total methylated (%) = (II + III + IV)/(I + II + III + IV) × 100. ^4^ Fully methylated = (II)/(I + II + III + IV) × 100. ^5^ Hemi-methylated = (III)/(I + II + III + IV) × 100. ^6^ Non-methylated = (I)/(I + II + III + IV) × 100.

**Table 3:** DNA methylation patterns in rice treated with gamma rays and DNA methyltransferase inhibitors detected by transposon methylation display using *p-SINE*1.

Band Type	M ^1^	H ^1^	Treatment Groups									
			Control	G100	G150	G250	A80	Z80	A80 + G100	A80 + Z150	Z80 + G100	Z80 + G150
I	1	1	97	148	162	164	166	167	254	266	287	299
II	1	0	53	54	52	55	51	49	44	43	44	41
III	0	1	16	45	46	48	66	64	115	121	121	130
IV	0	0	61	119	126	127	115	112	127	132	133	121
Total methylated (%) ^2,3^			57.27	59.56	58.03	58.38	58.29	57.40	52.96	52.67	50.94	49.41
Fully methylated (%) ^2,4^			23.35	14.75	13.47	13.96	12.81	12.50	8.15	7.65	7.52	6.94
Hemi-methylated (%) ^2,5^			7.05	12.30	11.92	12.18	16.58	16.33	21.30	21.53	20.68	22.00
Non-methylated (%) ^2,6^			42.73	40.44	41.97	41.62	41.71	42.60	47.04	47.33	49.06	50.59

^1^ M and H indicate the enzyme combinations *Mse*I/*Msp*I and *Mse*I/*Hpa*II; 0: band absent, 1: band present. ^2^ Methylation rate was calculated according to Choi et al. 2016 [42]. ^3^ Total methylated (%) = (II + III + IV)/(I + II + III + IV) × 100. ^4^ Fully methylated = (II)/(I + II + III + IV) × 100. ^5^ Hemi-methylated = (III)/(I + II + III + IV) × 100. ^6^ Non-methylated = (I)/(I + II + III + IV) × 100.

**Table 4:** Analysis of molecular variance (AMOVA) of methylation-sensitive amplified polymorphism (MSAP) and transposon methylation display (TMD) marker data.

Source	Df ^1^	SS ^2^	MS ^3^	Est. Var. ^4^	% ^5^	*P* (*rand* ≥ *data*) ^6^
**MSAP**						
Among groups	3	174.551	58.184	2.592	26%	0.001
Within groups	89	641.363	7.206	7.206	74%	
**TMD**						
Among groups	3	161.269	53.756	2.473	33%	0.001
Within groups	89	454.688	5.109	5.109	67%	
**MSAP + TMD**						
Among groups	3	335.821	111.940	5.065	29%	0.001
Within groups	89	1431.871	12.315	12.315	71%	

^1^ Degrees of freedom. ^2^ Sum of squared deviations. ^3^ Squared deviations. ^4^ Estimates of variance components. ^5^ Percentage of total variance contributed by each component. ^6^ Probability of a random value greater than or equal to the observed data value based on permutation test (*n* = 999).

## Supplementary Materials

The following are available online at https://www.mdpi.com/2223-7747/9/9/1088/s1. Figure S1: Delta *K* analysis for the identification of the optimal number of *K*. Plots for (**a**) methylation-sensitive amplified polymorphism (MSAP), (**b**) transposon methylation display (TMD), and (**c**) combined MSAP and TMD data. Table S1: The adapters and primers used in this study. Table S2: Primer combinations for methylation-sensitive amplified polymorphism (MSAP) and transposon methylation display (TMD) analyses.

## References

[1] Slotkin R., Martienssen R. (2007). Transposable elements and the epigenetic regulation of the genome. Nat. Rev. Genet..

[2] Zhang H., Lang Z., Zhu J. (2018). Dynamics and function of DNA methylation in plants. Nat. Rev. Mol. Cell Biol..

[3] Gonzalgo M., Jones P.A. (1997). Mutagenic and epigenetic effects of DNA methylation. Mut. Res..

[4] Venetsky A., Levy-Zamir A., Khasdan V., Domb K., Kashkush K. (2015). Structure and extent of DNA methylation-based epigenetic variation in wild emmer wheat (*T. turgidum* ssp. dicoccoides) populations. BMC Plant Biol..

[5] He X., Chen T., Zhu J. (2011). Regulation and function of DNA methylation in plants and animals. Cell Res..

[6] Bartels A., Han Q. (2018). Dynamic DNA methylation in plant growth and development. Int. J. Mol. Sci..

[7] Pecinka A., Liu C.H. (2014). Drugs for plant chromosome and chromatin research. Cytogenet. Genome Res..

[8] Griffin P.T., Niederhuth C.E., Schmitz R.J. (2016). A comparative analysis of 5-azacytidine- and zebularine-induced DNA demethylation. G3.

[9] Champion C., Guianvarc’h D., Sénamaud-Beaufort C., Jurkowska R.Z., Jeltsch A., Ponger L., Arimondo P.B., Guieysse-Peugeot A.L. (2010). mechanistic insights on the inhibition of c5 dna methyltransferases by zebularine. PLoS ONE.

[10] Kumpatla S.P., Teng W., Buchholz W.G., Hall T.C. (1997). Epigenetic transcriptional silencing and 5-azacytidine-mediated reactivation of a complex transgene in rice. Plant Physiol..

[11] Genger R.K., Peacock J.W., Dennis E.S., Finnegan J.E. (2003). Opposing effects of reduced DNA methylation on flowering time in *Arabidopsis thaliana*. Planta.

[12] Cheng Y.H., Peng X.Y., Yu Y.C., Sun Z.Y., Han L. (2019). The Effects of DNA Methylation inhibition on flower development in the dioecious plant *Salix viminalis*. Forests.

[13] Nowicka A., Tokarz B., Zwyrtková J., Dvořák Tomaštíková E., Procházková K., Ercan U., Finke A., Rozhon W., Poppenberger B., Otmar M. (2020). Comparative analysis of epigenetic inhibitors reveals different degrees of interference with transcriptional gene silencing and induction of DNA damage. Plant J..

[14] Flavell A.J., Pearce S.R., Kumar A. (1994). Plant transposable elements and the genome. Curr. Opin. Genet. Dev..

[15] Akakpo R., Carpentier M.C., Ie Hsing Y., Panaud O. (2020). The impact of transposable elements on the structure, evolution and function of the rice genome. New Phytol..

[16] Bennetzen J.L., Wang H. (2014). The contributions of transposable elements to the structure, function, and evolution of plant genomes. Annu. Rev. Plant Biol..

[17] Doolittle W.F., Sapienza C. (1980). Selfish genes, the phenotype paradigm and genome evolution. Nature.

[18] Orgel L.E., Crick F.H.C. (1980). Selfish DNA: The ultimate parasite. Nature.

[19] Volff J.N. (2006). Turning junk into gold: Domestication of transposable elements and the creation of new genes in eukaryotes. BioEssays.

[20] Wicker T., Sabot F., Hua-Van A., Bennetzen J.L., Capy P., Chalhoub B., Flavell A., Leroy P., Morgante M., Panaud O. (2007). A unified classification system for eukaryotic transposable elements. Nat. Rev. Genet..

[21] Kumar A., Bennetzen J.L. (1999). Plant retrotransposons. Annu. Rev. Genet..

[22] Song Q.X., Lu X., Li Q.T., Chen H., Hu X.Y., Ma B., Zhang W.K., Chen S.Y., Zhang J.S. (2013). Genome-Wide Analysis of DNA Methylation in Soybean. Mol. Plant.

[23] Zhang X., Yazaki J., Sundaresan A., Cokus S., Chan S.W., Chen H., Henderson I.R., Shinn P., Pellegrini M., Jacobsen S.E. (2006). Genome-wide high-resolution mapping and functional analysis of DNA methylation in *Arabidopsis*. Cell.

[24] Takata M., Kiyohara A., Takasu A., Kishima Y., Ohtsubo H., Sano Y. (2007). Rice transposable elements are characterized by various methylation environments in the genome. BMC Genom..

[25] Vos P., Hogers R., Bleeker M., Reijans M., van de Lee T., Hornes M., Frijters A., Pot J., Peleman J., Kuiper M. (1995). AFLP: A new technique for DNA fingerprinting. Nucleic Acids Res..

[26] Mondini L., Noorani A., Pagnotta M. (2009). Assessing plant genetic diversity by molecular tools. Diversity.

[27] Nadeem M.A., Nawaz M.A., Shahid M.Q., Doğan Y., Comertpay G., Yıldız M., Hatipoğlu R., Ahmad F., Alsaleh A., Labhane N. (2017). DNA molecular markers in plant breeding: Current status and recent advancements in genomic selection and genome editing. Biotechnol. Biotechnol. Equipment.

[28] Xu M., Li X., Korban S.S. (2000). AFLP-Based detection of DNA methylation. Plant Mol. Biol. Rep..

[29] Waalwijk C., Flavell R.A. (1978). DNA methylation at a CCGG sequence in the large intron of the rabbit beta-globin gene: Tissue-specific variations. Nucleic Acids Res..

[30] Khush G.S., Sasaki T., Moore G. (1997). Origin, dispersal, cultivation and variation of rice. Oryza: From Molecule to Plant.

[31] Viana V.E., Pegoraro C., Busanello C., de Oliveira A.C. (2019). Mutagenesis in Rice: The Basis for Breeding a New Super Plant. Front. Plant Sci..

[32] Moin M., Bakshi A., Saha A., Dutta M., Kirti P.B. (2017). Gain-of-function mutagenesis approaches in rice for functional genomics and improvement of crop productivity. Brief. Funct. Genom..

[33] Maekawa M., Hase Y., Shikazono N., Tanaka A. (2003). Induction of somatic instability in stable yellow leaf mutant of rice by ion beam irradiation. Nucl. Instrume. Methods Phys. Res. B.

[34] Kong X., Kasapis S., Bao J. (2015). Viscoelastic properties of starches and flours from two novel rice mutants induced by gamma irradiation. LWT-Food Sci. Technol..

[35] FAO/IAEA-MVD (2020). Food and Agriculture Organization of the United Nations/International Atomic Energy Agency—Mutant Variety Database. https://mvd.iaea.org/#!Search.

[36] Talebi A., Shahrokhifar B. (2012). Ethyl Methane Sulphonate (EMS) Induced mutagenesis in malaysian rice (cv. MR219) for lethal dose determination. Am. J. Plant Sci..

[37] Mo Y.J., Jeung J.U., Shin Y.S., Park C.S., Kang K.H., Kim B.K. (2013). Agronomic and genetic analysis of Suweon 542, a rice floury mutant line suitable for dry milling. Rice.

[38] Satoh H., Matsusaka H., Kumamaru T. (2010). Use of N-methyl-N-nitrosourea treatment of fertilized egg cells for saturation mutagenesis of rice. Breed. Sci..

[39] Theerawitaya C., Triwitayakorn K., Kirdmanee C., Smith D., Supaibulwatana K. (2011). Genetic variations associated with salt tolerance detected in mutants of KDML105 (*Oryza sativa* L. spp. *indica*) rice. Aust. J. Crop. Sci..

[40] Singh S., Sharma R., Singh P., Chakravarti S. (2016). Gamma ray and EMS induced effectiveness and efficiency of chlorophyll mutations in aromatic rice (*Oryza sativa* L.). Ecoscan.

[41] Manikandan V., Vanniarajan C. (2017). Induced macromutational spectrum and frequency of viable mutants in M_2_ generation of rice (*Oryza sativa* L.). Int. J. Curr. Microbiol. Appl. Sci..

[42] Choi J.Y., Roy N.S., Park K.C., Kim N.S. (2016). Comparison of molecular genetic utilities of TD, AFLP, and MSAP among the accessions of *japonica*, *indica*, and Tongil of *Oryza sativa* L.. Gen. Genom..

[43] Foust C.M., Preite V., Schrey A.W., Alvarez M., Robertson M.H., Verhoeven K.J., Richards C.L. (2016). Genetic and epigenetic differences associated with environmental gradients in replicate populations of two salt marsh perennials. Mol. Ecol..

[44] Guarino F., Cicatelli A., Brundu G., Improta G., Triassi M., Castiglione S. (2019). The use of MSAP reveals epigenetic diversity of the invasive clonal populations of *Arundo donax* L.. PLoS ONE.

[45] Walbot V. (1988). Reactivation of the Mutator transposable element system following gamma irradiation of seed. Mol. Gen. Genet..

[46] Yu H., Zhao J., Xu J., Li X., Zhang F., Wang Y., Carr C., Zhang J., Zhang G. (2011). Detection of changes in DNA methylation induced by low-energy ion implantation in *Arabidopsis thaliana*. Radiat. Res..

[47] Pikaard C., Mittelsten Scheid O. (2014). Epigenetic regulation in plants. Cold Spring Harb. Perspect. Biol..

[48] Ohtsubo H., Tsuchimoto S., Xu J., Cheng C., Koudo M., Kurata N., Ohtsubo E., Hirano H.-Y., Sano Y., Hirai A., Sasaki T. (2008). Rice retroposon, *p-SINE*, and its use for classification and identification of *Oryza* species. Rice Biology in the Genomics Era.

[49] Motohashi R., Mochizuki K., Ohtsubo H., Ohtsubo E. (1997). Structures and distribution of *p-SINE1* members in rice genomes. Theor. Appl. Genet..

[50] Tirnaz S., Batley J. (2019). Epigenetics: Potentials and challenges in crop breeding. Mol. Plant.

[51] Gallusci P., Dai Z., Génard M., Gauffretau A., Leblanc-Fournier N., Richard-Molard C., Vile D., Brunel-Muguet S. (2017). Epigenetics for plant improvement: Current knowledge and modeling avenues. Trends Plant Sci..

[52] Reyna-López G.E., Simpson J., Ruiz-Herrera J. (1997). Differences in DNA methylation patterns are detectable during the dimorphic transition of fungi by amplification of restriction polymorphisms. Mol. Gen. Genet..

[53] Yeh F., Yang R., Boyle T. (1999). POPGENE Version 1.31, Microsoft Windows-Based Freeware for Population Genetic Analysis: Quick User Guide.

[54] Peakall R., Smouse P.E. (2012). GenAlEx 6.5: Genetic analysis in Excel. Population genetic software for teaching and research-an update. Bioinformatics.

[55] Liu K., Muse S. (2005). PowerMarker: An integrated analysis environment for genetic marker analysis. Bioinformatics.

[56] Kumar S., Stecher G., Li M., Knyaz C., Tamura K. (2018). MEGA X: Molecular evolutionary genetics analysis across computing platforms. Mol. Biol. Evol..

[57] Pritchard J.K., Stephens M., Donnelly P. (2000). Inference of population structure using multilocus genotype data. Genetics.

[58] Earl D.A., vonHoldt B.M. (2012). STRUCTURE HARVESTER: A website and program for visualizing STRUCTURE output and implementing the Evanno method. Conserv. Genet. Resour..

