# Supplementary material for: Epigenetic Variation Induced by Gamma Rays, DNA Methyltransferase Inhibitors, and Their Combination in Rice
Source: Plants (Basel). 2020 Aug 24;9(9):1088. doi: 10.3390/plants9091088 (PMC7570246; doi:10.3390/plants9091088)
Supplement: Supplementary file 1 [file plants-09-01088-s001.zip › Supplementary tables.docx]

**Table S1.** The adapters and primers used in this study.

| **Name** | **Sequence (5′ → 3′)** | **Note** |
| --- | --- | --- |
| *Mse*I |  |  |
| *Mse*I-A1 | GACGATGAGTCCTGAG | Adapter |
| *Mse*I-A2 | TACTCAGGACTCAT | Adapter |
| *Mse*I-0 | GACGATGAGTCCTGAGTAA | Pre-amplification |
| *Mse*I-CAT | GACGATGAGTCCTGAGTAACAT | Selective amplification |
| *Mse*I-CTA | GACGATGAGTCCTGAGTAACTA | Selective amplification |
| *Mse*I-GAT | GACGATGAGTCCTGAGTAAGAT | Selective amplification |
| *Mse*I-GAG | GACGATGAGTCCTGAGTAAGAG | Selective amplification |
| *Msp*I/*Hpa*II |  |  |
| *MH*-A1 | GATCATGAGTCCTGCT | Adapter |
| *MH*-A2 | AGTACTCAGGACGAGC | Adapter |
| *MH*-0 | ATCATGAGTCCTGCTCGG | Pre-amplification |
| *MH*-AAG | ATCATGAGTCCTGCTCGGAAG | Selective amplification |
| *MH*-TAG | ATCATGAGTCCTGCTCGGTAG | Selective amplification |
| *MH*-TCA | ATCATGAGTCCTGCTCGGTCA | Selective amplification |
| *MH*-CTA | ATCATGAGTCCTGCTCGGCTA | Selective amplification |
| Transposon specific primer |  |  |
| *p-SINE*1 | TGTGGAGCTAGCCGGAGAC | Specific to *p-SINE*1 |

**Table S2.** Primer combinations for methylation-sensitive amplified polymorphism (MSAP) and transposon methylation display (TMD) analyses.

| **Marker system** | **Primer combination** |
| --- | --- |
| MSAP | *Mse*I-CAT + *MH*-AAG |
|  | *Mse*I-CAT + *MH*-TAG |
|  | *Mse*I-CTA + *MH*-TCA |
|  | *Mse*I-GAT + *MH*-TAG |
|  | *Mse*I-GAT + *MH*-CTA |
|  | *Mse*I-GAG + *MH*-CTA |
| TMD | *p-SINE*1 + *MH*-AAG |
|  | *p-SINE*1 + *MH*-TAG |
|  | *p-SINE*1 + *MH*-TCA |
|  | *p-SINE*1 + *MH*-CTA |
